# Predicting direct healthcare costs of general practitioner–guided care in patients with musculoskeletal complaints

**DOI:** 10.1097/j.pain.0000000000003028

**Published:** 2023-08-15

**Authors:** Sylvia Pellekooren, Ângela J. Ben, Johanna M. van Dongen, Annelies L. Pool-Goudzwaard, Maurits W. van Tulder, Jesse M. van den Berg, Raymond W.J.G. Ostelo

**Affiliations:** a Department Human Movement Sciences, Faculty of Behavioral and Movement Sciences, Amsterdam Movement Sciences Research Institute, Amsterdam, the Netherlands; b Department of Health Sciences, Faculty of Science, Vrije Universiteit Amsterdam, Amsterdam Movement Sciences Research Institute Amsterdam, the Netherlands; c Department of Health Sciences, Faculty of Science, Vrije Universiteit Amsterdam, Amsterdam Public Health Research Institute, Amsterdam, the Netherlands; d Somt University of Physiotherapy, Amersfoort, the Netherlands; e PHARMO Institute for Drug Outcomes Research, Utrecht, the Netherlands; f Department of General Practice, Amsterdam UMC Location Vrije Universiteit Amsterdam, Amsterdam, the Netherlands; g Amsterdam Public Health, Health Behaviors and Chronic Diseases, Amsterdam, the Netherlands; h Department of Epidemiology and Biostatistics, Amsterdam UMC, Vrije Universiteit Amsterdam, Amsterdam Movement Sciences Research Institute, Amsterdam, the Netherlands

**Keywords:** Musculoskeletal complaints, Primary care, General practice, Healthcare costs

## Abstract

Information on healthcare utilization and costs of general practitioner (GP)–guided care in patients with musculoskeletal complaints is important for keeping healthcare affordable and accessible. A registry-based study was performed to describe healthcare utilization and costs of GP-guided care in patients with musculoskeletal complaints and to predict having higher direct healthcare costs. Healthcare costs of GP-guided care included all healthcare resources used by patients due to a musculoskeletal condition in 2018. Data were extracted from the database with a 1-year follow-up and descriptively analyzed. A general linear model was developed to predict having higher direct healthcare costs. In total, 403,719 patients were included, of whom 92% only received a single consultation. The number of referrals varied across the different types of complaints. Total annual direct healthcare costs amounted to €39,180,531, of which a key cost driver was referrals. Primary care consultations accounted for the largest part of referral-related costs. For all musculoskeletal conditions combined, the mean annual direct healthcare cost per patient was €97 (SEM = €0.18). Older age, being a woman, low socioeconomic status, spine complaints, high number of musculoskeletal diagnoses, and a high comorbidity score were predictive of having higher direct healthcare costs and explained 0.7% of the variance. This study showed that mean annual direct healthcare costs of GP-guided care in patients with musculoskeletal conditions were relatively low and did not differ considerably across conditions. The predictive model explained a negligible part of the variance in costs. Thus, it is unclear which factors do predict high direct healthcare costs in this population.

## Abstract 2

Supplemental Digital Content is Available in the Text.

Mean direct healthcare costs of general practitioner–guided care in patients with musculoskeletal conditions were relatively low. It is unclear which factors predict having higher direct healthcare costs.

## 1. Introduction

Most patients with musculoskeletal conditions are treated in a primary care setting. Physiotherapists and general practitioners (GPs) are often the first point of contact. In the Netherlands, 14.6% of all contacts with a GP are related to musculoskeletal conditions.^12^ Besides representing a large group of patients in primary care, patients with musculoskeletal conditions also contribute to high healthcare costs. In 2017, the total healthcare costs of musculoskeletal conditions in the Netherlands—including primary, secondary, and tertiary care—were estimated at 6.3 billion euros, equaling about 7.5% of the total Dutch healthcare budget.^29^ According to a report by the Dutch Ministry of Health, Welfare, and Sports, the total costs of musculoskeletal complaints in general practices amount to 342.5 million euros, which is 8.8% of the total GP costs.^29^ Estimations based on demographic development (ie, population growth and aging) and changing healthcare use (ie, medical technology and increased welfare) forecast that the healthcare costs of musculoskeletal conditions will increase during the upcoming decades, with an average of 2.85% per year.^30^ Research also indicates that a considerable part of these costs is attributable to a relatively small group of patients, so-called high-cost users.^18,36^ Previous studies provided insight into the resource use and costs of patients with musculoskeletal conditions in a broad range of settings, as well as predictive factors of having high(er) costs.^3,18,36^ However, previous studies assessing the costs of musculoskeletal conditions in general practices either only presented costs at the aggregate level and not per musculoskeletal condition separately or reported on patients with low back pain only. The aim of this study was to describe healthcare utilization and costs of GP-guided care for patients with various musculoskeletal complaints separately and to predict higher direct healthcare costs in GP-guided care for patients with musculoskeletal complaints.

## 2. Methods

### 2.1. Setting and population

The Dutch healthcare system is characterized by a demand-driven system with regulated competition and elements of both public and private insurance. All Dutch residents are mandated to have a basic health insurance package, which includes—among others—a standard package of care provided by GPs, with GP consultations being fully reimbursed.^21^ Most of all healthcare pathways start with consulting a GP, as in the Dutch healthcare system, patients are registered at one GP only, who is the gatekeeper for patients' referral to primary and secondary healthcare facilities.^7^ Patients with musculoskeletal complaints, however, can also consult a physiotherapist directly through direct access physiotherapy. In 2018, 68% of patients consulted a physiotherapist without a GP referral.^35^ In describing healthcare utilization and costs for patients with musculoskeletal complaints, consultations related to direct access physiotherapy were not included as this care is guided by physiotherapists and not by GPs. Other allied healthcare professionals are only accessible through a GP referral. One full-time GP provides medical care for approximately 2350 patients.^7^

### 2.2. Source of data

Data were used from the PHARMO Database Network.^27^ This database contains longitudinal real-world patient data that are retrieved, among others, from GPs' electronic patient files, pharmacies, and laboratories throughout the Netherlands, and includes electronic medical records of 2,118,603 Dutch adult patients, which is 13% of all adult patients registered with a GP practice in the Netherlands.^11^ Data were recorded as part of routine clinical practice at GP offices and included prospectively collected anonymous patient-level data on consultations, medication, and referrals by GPs. This information was extracted from problem lists and journal text. Patients of 18 years and older were included in this study when an International Classification of Primary Care (ICPC) code^17^ that represents a musculoskeletal complaint was assigned in 2018. Follow-up data had to be available for a minimum duration of 1 year after allocating the ICPC code (ie, index date). An overview of included ICPC codes is presented in Appendix I (available as supplemental digital content at http://links.lww.com/PAIN/B906). No ethical approval was needed because this study analyzed already existing completely anonymous data.

### 2.3. Healthcare utilization

Healthcare utilization of GP-guided care was defined as all healthcare resources used by patients due to a musculoskeletal condition guided by a GP and included GP care itself (office-based consultations, home visits, consultations by phone or email, and consultations by a GP-based nurse specialist or physician assistant), referrals to other healthcare providers (to primary care—i.e., physiotherapy, manual therapy, occupational therapy, exercise therapy, dietetics, mental health services, and orthopedic technician; referrals to secondary care—ie, consultation medical specialist; and referrals to diagnostic imaging—ie, echography, X-ray, CT, and MRI), and prescribed medication. Referrals to primary care involved treatment episodes with primary care givers (eg, physical therapist), whereas referrals to secondary care only involved a single consultation by a secondary care provider (eg, medical specialist). Secondary care referrals did not include any follow-up appointments, examinations, surgery, possible admissions, or cost related to the provision of specialized treatment because this care is guided by medical specialists and not by GPs and was therefore not included in this data set. Although the patients' actual attendance to referral visits could not be confirmed, it was assumed that all patients attended their referral appointment because secondary care in the Netherlands is only accessible through a referral by the GP, is fully reimbursed by the basic healthcare insurance which is mandatory for all Dutch residents, and no-show rates in the Netherlands are relatively low. Referrals that were not related to a musculoskeletal complaint (eg, an ophthalmologist) were excluded from further analysis. Prescribed medication was classified according to the Anatomical Therapeutic Chemical (ATC) classification system.^37^

### 2.4. Direct healthcare costs

Direct healthcare costs were defined as costs of the healthcare utilization described above. GP care was valued using Dutch standard prices derived from the Dutch guidelines for costing studies.^13,22^ For referrals to primary care providers, the patients' number of contacts could not be retrieved from the database and were estimated based on national estimates of the average number of contacts for a broad range of care providers (eg, physiotherapy and exercise therapy).^11,24^ For social work and primary care–based psychologists, the number of contacts was based on data from the Netherlands Institute of Mental Health and Addiction.^33^ Dutch standard prices were used to value healthcare utilization.^13,22^ Medication use was valued using prices published by the National Health Care Institute.^23^ All costs were expressed in euros 2021. The time horizon for costs was 1 year. Refer to Table 1 for more details.

**Table 1:** Costing details, consultations, referrals, and medication.

	Unit cost (€)	Number of units	Total cost (€)
Consultations*			
Office-based consult	36.04	1	36.04
Home visit	54.60	1	54.60
Consultation by phone or email	18.56	1	18.56
Consultation by general practice–based nurse specialists	18.56	1	18.56
Referrals*			
Primary care			
Physiotherapy	36.04	7.5	270.3
Occupational therapy	36.04	8.1	219.92
Manual therapy	36.04	7.5	270.3
Exercise therapy	37.13	10.5	389.87
Podotherapy	279.23	1	279.23
Medical devices	1050	1	1050
Dietetics	32.41	3.2	103.71
Psychologist	100.81	8	806.48
Social worker	70.98	8	567.84
Secondary care			
Consultation medical specialist	132.68	1	132.68
Imaging			
Radiological	170.95	1	170.95
Echography	93.60	1	93.60
Medication†			
PCM	0.07	1	0.07
NSAIDs	0.08	1	0.08
Opioids weak	0.06	1	0.06
Opioids strong	2.04	1	2.04
Coanalgesic	0.05	1	0.05
Injections‡	4.77	1	4.77

Costs are presented in euros 2021.

* Unit costs are based on Dutch guidelines for costing studies.^13,22^

† Unit costs are based on prices published by the National Health Care Institute.^23^

‡ The GP consultation during which the injection was administered is counted separately under consultation.

### 2.5. Predictive factors

Based on previous literature, a set of potential predictive factors were used to predict higher direct healthcare costs,^18,37^ but only predictive factors that were registered in the database could be included. Factors included were age (years), sex (male/female), region of complaint (ie, lower extremity, upper extremity, and spine), type of complaint (ie, based on guidelines for general practitioners^8^), the Chronic Disease Score (ie, overall score retrieved by an algorithm for comorbidities which is based on assigned ICPC codes and prescribed medication), cardiovascular disease (yes/no), diabetes mellitus (yes/no), smoking (yes/no), obesity (yes/no), the total number of musculoskeletal diagnoses, depression (yes/no), and neighborhood socioeconomic status (SES) (ie, low, middle, or high).^15,33^ Some of the variables included were generated based on other ICPC codes assigned to the patients. To illustrate, cardiovascular disease (yes/no), diabetes mellitus (yes/no), smoking (yes/no), and obesity (yes/no) were based on whether patients received ICPC codes for these conditions and were considered from the time of inception of the database (ie, 2008) up until the index date. For depression, a similar approach was used, but only the 6-month period before the index date was considered. The Chronic Disease Score is defined as a set of scoring rules that render a score between 0 and 41, reflecting chronic disease severity based on a single drug or combinations of drugs. The CDS is associated with subsequent-year hospitalization and mortality: a CDS of 7 or greater is associated with a 5-fold increase in the risk of hospitalization and a 10-fold increase in the risk of dying.^6,26^ Further details on variables region of complaint and type of complaint are described in Appendix I (available as supplemental digital content at http://links.lww.com/PAIN/B906). Further details on the variables SES, cardiovascular disease, diabetes mellitus, smoking, and obesity are shown in Appendix II (available as supplemental digital content at http://links.lww.com/PAIN/B906). The total number of musculoskeletal complaints was based on the number of musculoskeletal-related ICPC codes that patients received during the 6-month period before the index date.

### 2.6. Statistical analysis

Seven percent of all patients had one or more missing values. These were imputed using the Multivariate Imputation by Chained Equation (MICE) package.^4,37^ The MICE framework uses predictive mean matching (PMM) to generate several data sets with complete data randomly sampled from the observed data after matching observations with missing values with those with complete observed data (referred to as donor observations). Predictive mean matching assumes that the distribution of the missing data is the same as the observed data and avoids imputing implausible values (negative costs).^4,37^ Five complete data sets were generated using an imputation model that included ICPC code, sex, age, SES, the total number of musculoskeletal complaints, Chronic Disease Score, cardiovascular disease, depression, obesity, smoking, type of referral, type of medication, type of GP consultation, and observation linked to the episode of interest (ie, the GP consultation, referral, or prescribed medication is related to the musculoskeletal complaint) (yes/no). After data imputation, healthcare utilization and costs of GP-guided care were descriptively analyzed using means and SEMs (continuous variables) and counts and percentages (dichotomous and categorical variables).

For the descriptive analyses, healthcare utilization and costs were presented for the entire study population, as well as for patients who belonged to 1 of the 4 cost quartiles and those who belonged to the top 5% of high-cost users. Results were presented for all musculoskeletal conditions combined, by region of complaint (ie, spine, lower extremity, and upper extremity) and for the 5 most common types of complaint based on Dutch GP guidelines (ie, hand/wrist complaints, knee complaints, low back pain, low back pain with radiation, and shoulder complaints).^8^ More detailed information on ICPC codes included per region and per type of complaint is presented in Appendix I (available as supplemental digital content at http://links.lww.com/PAIN/B906).

The dependent variable in the regression analysis was having higher direct healthcare costs, which was included as a continuous outcome. To predict higher direct healthcare costs of GP-guided care, each of the 5 complete data sets was analyzed separately as outlined below, after which results were pooled using Rubin rules.^4,20^ Total direct healthcare costs were regressed on the possible predictive factors using a general linear model (GLM) with a gamma distribution and an identity link. A GLM was chosen because of the skewed distribution of healthcare costs.^1^ Before the analyses, assumptions of a GLM with a gamma distribution were checked (ie, normally distributed residual variance, homoscedasticity, influential cases, and outliers). The model was trained and evaluated using the caret package.^16^ K-fold cross-validation was used to internally validate the model.^2^ Predictors were selected using a bidirectional stepwise selection procedure,^31^ using the Akaike information criterion (ie, the trade-off between the goodness of fit of the model and the simplicity of the model),^5^ with a 5% significance level. Stepwise selection combines the elements of forward and backward selection by sequentially adding variables, based on the most contributing predictors, and omitting variables that no longer provide an improvement in the model fit after adding a new variable to the model. The final model only included case-mix variables that increased the predictive value. The overall performance of the model was assessed using the RMSE (ie, the absolute fit of the model) and the adjusted R2 (ie, the relative fit of the model). Analyses were conducted in R (version 4.0.0).

## 3. Results

In total, 403,719 patients were included. The mean age was 53 (SD = 18) years and 227,101 patients (56%) were female. The most common region of complaints was the lower extremities (28%), and the most common type of complaint was shoulder complaints 34,579 (9%). Refer to Table 2 for more characteristics and Appendices III and IV (available as supplemental digital content at http://links.lww.com/PAIN/B906) for more details for region and type of complaint.

**Table 2:** Baseline characteristic of overall musculoskeletal complaints.

	Overall (n = 403,719)	Q1/Q2* (n = 207,625)	Q3 (n = 95,298)	Q4 (n = 100,796)	Top 5% HCU (n = 20,187)
Sex: female (yes; n, %)	227,101 (56.3)	114,496 (55.1)	53,522 (56.2)	59,083 (58.6)	12,147 (60.2)
Age (mean, SD)	52.54 (18.31)	51.81 (18.41)	52.63 (18.52)	53.95 (17.82)	54.93 (17.55)
Comorbidities					
Chronic Disease Score (mean, SD)	2.78 (3.71)	2.56 (3.60)	2.92 (3.76)	3.09 (3.87)	3.20 (3.87)
Number of musculoskeletal diagnoses (mean, SD)	0.08 (0.31)	0.08 (0.31)	0.08 (0.31)	0.08 (0.33)	0.09 (0.33)
Depression (yes; n, %)	2225 (0.6)	1127 (0.5)	551 (0.6)	547 (0.5)	102 (0.5)
Obese (yes; n, %)	1314 (0.3)	593 (0.3)	364 (0.4)	357 (0.4)	86 (0.4)
Smoking (yes; n, %)	1726 (0.4)	807 (0.4)	468 (0.5)	451 (0.4)	91 (0.5)
Cardiovascular (yes; n, %)	112,979 (28.0)	54,923 (26.5)	27,706 (29.1)	30,350 (30.1)	6384 (31.6)
Diabetes (yes; n, %)	22,758 (5.6)	10,718 (5.2)	5696 (6.0)	6344 (6.3)	1323 (6.6)
Socioeconomic status					
Low (n, %)	146,820 (36.4)	78,052 (37.6)	30,528 (32.0)	38,240 (37.9)	7832 (38.8)
Middle (n, %)	132,624 (32.9)	70,775 (34.1)	31,728 (33.3)	30,121 (29.9)	5767 (28.6)
High (n, %)	124,275 (30.8)	58,798 (28.3)	33,042 (34.7)	32,435 (32.2)	6588 (32.6)
Region of complaint					
Spine (n, %)	74,553 (18.5)	36,879 (17.8)	18,156 (19.1)	19,518 (19.4)	5028 (24.9)
Upper extremity (n, %)	109,202 (27.0)	57,007 (27.5)	26,039 (27.3)	26,156 (25.9)	4737 (23.5)
Lower extremity (n, %)	113,449 (28.1)	55,701 (26.8)	24,875 (26.1)	32,873 (32.6)	6543 (32.4)
Other (n, %)	106,515 (26.4)	58,038 (28.0)	26,228 (27.5)	22,249 (22.1)	3879 (19.2)
Type of complaint†					
Ankle (n, %)	7284 (1.8)	4267 (2.1)	1628 (1.7)	1389 (1.4)	213 (1.1)
Arthritis (n, %)	721 (0.2)	223 (0.1)	223 (0.2)	275 (0.3)	69 (0.3)
Epicondylitis (n, %)	6827 (1.7)	4383 (2.1)	1498 (1.6)	946 (0.9)	184 (0.9)
Fracture (n, %)	1245 (0.3)	687 (0.3)	358 (0.4)	200 (0.2)	30 (0.1)
Hand/wrist (n, %)	23,107 (5.7)	11,884 (5.7)	5518 (5.8)	5705 (5.7)	866 (4.3)
Knee (n, %)	12,308 (3.0)	5392 (2.6)	3462 (3.6)	3454 (3.4)	870 (4.3)
Knee acute (n, %)	6460 (1.6)	2761 (1.3)	1511 (1.6)	2188 (2.2)	404 (2.0)
Low back pain (n, %)	20,976 (5.2)	10,750 (5.2)	5318 (5.6)	4908 (4.9)	1238 (6.1)
Low back pain radicular (n, %)	9989 (2.5)	3554 (1.7)	2782 (2.9)	3653 (3.6)	1040 (5.2)
Rheumatoid arthritis (n, %)	520 (0.1)	101 (0.0)	90 (0.1)	329 (0.3)	146 (0.7)
Shoulder (n, %)	34,579 (8.6)	14,895 (7.2)	9732 (10.2)	9952 (9.9)	2374 (11.8)
Others	124,016 (30.7)	58,897 (28.3)	32,120 (33.7)	32,999 (32.8)	7434 (36.8)

Q, quartile; HCU, high-cost users.

Chronic Disease Score: range 0 to 41. A score of 7 or greater is associated with a 5-fold increase in risk of hospitalization and a 10-fold increase in risk of dying.

* Q1and Q2 are merged as many patients had the same cost and were therefore hard to disguise.

† Type of complaint is based on general practitioners' guidelines.^7^

### 3.1. Healthcare utilization

The most common type of GP-guided care was office-based consultations (93%). Of all patients, 92% received a single consultation only, and 21% received one or more referrals (of which 41% were to primary caregivers, 43% to a medical specialist, and 7% to diagnostic imaging). Of all referrals to a primary care provider, 23% were physiotherapy referrals, 3% were podiatry referrals, and 1% were exercise therapy referrals. Medication was prescribed to 17% of patients, with NSAIDs being most prescribed (41%). The number of referrals varied widely across the different types of complaints. For example, of all patients who consulted the GP because of low back pain, 20% received a referral, of which 27% were to a secondary care facility. In case of hand or wrist complaints, 22% of patients received a referral, of which 62% made was to a secondary care facility. Refer to Table 3 for more details for region and type of complaint.

**Table 3:** Healthcare utilization during 1-year follow-up.

	Overall MSK (n = 403,719)	Region spine (n = 74,553)	Region upper extremity (n = 109,202)	Region lower extremity (n = 113,449)	Hand/wrist (n = 23,107)	Knee (n = 12,308)	LBP (n = 20,976)	LBP radicular pain (n = 9989)	Shoulder (n = 34,579)
Healthcare utilization									
One-off consultation (yes; n, %)	372,743 (92.3)	67,938 (91.1)	102,499 (93.9)	104,144 (91.8)	21,964 (95.1)	11,231 (91.2)	19,229 (91.7)	8856 (88.7)	31,896 (92.2)
Medication prescribed (yes; n, %)	68,867 (17.1)	18,169 (24.4)	22,554 (20.7)	13,568 (12.0)	4542 (19.7)	3833 (31.1)	5908 (28.2)	3502 (35.1)	11,943 (34.5)
Referral (yes; n, %)	85,042(21.1)	16,132 (21.6)	22,880 (21.0)	28,103 (24.8)	5156 (22.3)	2900 (23.6)	4108 (19.6)	3056 (30.6)	8523 (24.6)
Referrals									
Total number of referrals (n)	117,980	22,436	30,074	38,495	6579	4206	5386	4492	11,422
Referrals to primary care* (n, %)	48,424 (41.1)	12,533 (55.9)	12,195 (40.6)	14,451 (37.5)	1942 (29.5)	1511 (35.9)	3468 (64.4)	2058 (45.8)	6105 (53.5)
Referrals to secondary care (n, %)	50,636 (42.9)	7594 (33.8)	14,115 (46.9)	18,214 (47.3)	4089 (62.2)	2299 (54.7)	1468 (27.3)	2045 (45.5)	4415 (38.7)
Referrals for imaging (n, %)	8501 (7.2)	742 (3.3)	1952 (6.5)	3088 (8.0)	264 (4.0)	201 (4.8)	165 (3.0)	116 (2.6)	395 (3.4)
Other referrals† (n, %)	10,419 (8.8)	1567 (7.0)	1812 (6.0)	2742 (7.2)	284 (4.3)	195 (4.6)	285 (5.3)	273 (6.1)	507 (4.4)

MSK, musculoskeletal complaint; LBP, low back pain.

* Includes referral to occupational therapy, physiotherapy, exercise therapy, manual therapy, podotherapy and orthopedic devices, and dietetic.

† Includes referrals to non–pre-specified disciplines that deemed to be not clinically relevant as it is possible that patients consult their GP for more than one complaint during the same consultation, whereas the consultation was assigned to an ICPC code related to the musculoskeletal complaint.

### 3.2. Direct healthcare costs

In the complete study sample, the 1-year mean total direct healthcare cost of GP-guided care per patient was €97 (SEM = €0.18). The 1-year mean cost per patient for GP consultations and referrals (ie, cost generated by the referral) was €43 (SEM = €0.03) and €53 (SEM = €0.18), respectively. Primary care referrals were associated with the highest cost average per patient (€33; SEM = €0.14), followed by secondary care referrals (€17; SEM = €0.07), and diagnostic imaging (€4; SEM = €0.03). For prescribed medication, the 1-year mean cost per patient was €1 (SEM = €0.02). Among patients who were referred, 1-year average primary care and secondary care costs were €303 (SEM = €0.51) and €155 (SEM = €0.30). Among patients referred to diagnostic imaging, the 1-year average imaging cost was €177 (SEM = €0.36). More detailed information on the 1-year mean healthcare cost of GP-guided care per patient is shown in Table 4.

**TABLE 4:** Healthcare costs per patient for overall MSK during 1-year follow-up.

	Overall (n = 403,719)	Q1/Q2* (n = 207,625)	Q3 (n = 95,298)	Q4 (n = 100,796)	Top 5% HCU (n = 20,187)
Consultation costs (mean, SEM)†	43 (0.03)	36 (0.00)	50 (0.06)	51 (0.12)	55 (0.34)
Medication costs (mean, SEM)	1 (0.02)	0.00 (0.00)	3 (0.01)	3 (0.08)	6 (1.12)
Referrals primary care costs (mean, SEM)	33 (0.14)	0.00 (0.00)	2 (0.03)	130 (0.42)	279 (1.10)
Referrals secondary care costs (mean, SEM)	17 (0.07)	0.00 (0.00)	4 (0.04)	63 (0.22)	106 (0.74)
Referrals imaging costs (mean, SEM)	4 (0.03)	0.00 (0.00)	1 (0.02)	14 (0.11)	20 (0.32)
Total referrals costs (mean, SEM)	53 (0.18)	0.00 (0.00)	7 (0.05)	206 (0.44)	405 (1.11)
Total cost (mean, SEM)	97 (0.18)	36 (0.08)	58 (0.05)	259 (0.44)	466 (1.12)

Q: quartile, SEM: standard error of the mean, HCU: high-cost users.

Costs are presented in euros 2021.

* Q1and Q2 are merged as many patients had the same cost and were therefore hard to distinguish.

† Consultation cost refer to GP consultations (office-based consultations, home visits, consultations by phone/email, and consultations by a GP-based nurse specialist or physician assistant).

Total 1-year mean costs were similar among the different regions and types of complaints except for low back pain with radiation, of which the 1-year average cost per patient was €137 (SEM = €1.58). The 1-year mean total direct healthcare cost for high-cost users (ie, top 5%) was €466 (SEM €1.12), with referrals to primary caregivers being the largest cost driver. More detailed information on costs per region and type of complaint is shown in Appendix V (available as supplemental digital content at http://links.lww.com/PAIN/B906).

In the complete study sample (n = 403,719), the 1-year total direct healthcare costs of GP-guided care amounted to €39,180,531. High-cost users were responsible for 24% of these costs (ie, €9,406,681). More detailed information on 1-year total direct healthcare costs of GP-guided care per region and per type of complaint is shown Table 5.

**Table 5:** OVERALL total healthcare costs per region and type of complaint.

	Total healthcare costs (mean, SEM)	Total costs consultation (mean, SEM)	Total cost referral primary care givers (mean, SEM)	Total cost referral medical specialist (mean, SEM)	Total cost imaging (mean, SEM)	Total cost referral (mean, SEM)	Total cost medication (mean, SEM)
Overall	39,180,531 (0.18)	17,313,539 (0.03)	13,269,305 (0.03)	6,703,684 (0.14)	1,439,536 (0.07)	21,412,524 (0.18)	466.684 (0.02)
Region							
Spine	8,027,792 (0.50)	3,283,034 (0.08)	3,466,607 (0.39)	998,417 (0.15)	126,503 (0.05)	4,591,527 (0.47)	154,139 (0.08)
Upper extremity	10,156,463 (0.33)	4,508,261 (0.05)	3,315,373 (0.25)	1,864,738 (0.13)	331,301 (0.05)	5,511,412 (0.32)	134,221 (0.04)
Lower extremity	11,871,861 (0.36)	4,913,076 (0.07)	3,941,983 (0.26)	2,407,213 (0.15)	525,876 (0.06)	6,875,072 (0.35)	91,193 (0.03)
Type of complaint							
Hand/wrist	2,079,571 (0.66)	936,386 (0.11)	527,759 (0.46)	539,132 (0.34)	44,926 (0.08)	1,111,816 (0.65)	29,432 (0.05)
Knee	1,315,833 (1.16)	548,362 (0.23)	406,809 (0.80)	299,247 (0.51)	35,113 (0.14)	741,169 (1.11)	26,390 (0.07)
Low back pain	2,111,707 (0.88)	896,601 (0.13)	955,999 (0.74)	194,005 (0.22)	28,651 (0.09)	1,178,656 (0.85)	37,468 (0.11)
Low back pain radicular	1,371,777 (1.58)	464,041 (0.26)	576,642 (1.18)	264,590 (0.60)	21,129 (0.14)	862,362 (1.49)	40,842 (0.34)
Shoulder	3,859,345 (0.71)	1,476,802 (0.11)	1,661,514 (0.57)	581,112 (0.24)	65,816 (0.08)	2,308,442 (0.69)	74,332 (0.11)

SEM, standard error of the mean.

Costs are presented in euros 2021.

### 3.3. Predictive factors

Predictive factors for having higher direct healthcare costs were older age, being a woman, low socioeconomic status, spine complaints, a high number of musculoskeletal diagnoses, and a high comorbidity score. The model explained 0.7% (*R*^2^) of the variation in the outcome (ie, higher direct healthcare costs) and the absolute fit (RMSE) was 127.8. More details on the regression model are presented in Appendix VI (available as supplemental digital content at http://links.lww.com/PAIN/B906).

## 4. Discussion

### 4.1. Main findings

Most patients visiting a GP with a musculoskeletal complaint only received a single consultation. The 1-year mean annual direct healthcare costs per patient were relatively similar across conditions, except for low back pain with radiation. The total annual direct healthcare costs in the complete study sample amounted to €39,180,531. The key cost driver consisted of referrals, with total costs of €21,412,524, more than half of which was for referrals to primary care providers. The top 5% of high-cost users were responsible for 24% of the costs. Older age, being a woman, low socioeconomic status, spine complaints, a high number of musculoskeletal diagnoses, and a high comorbidity score were predictive factors for having higher direct healthcare costs, but only explained 0.7% of the variance.

### 4.2. Comparison with literature

The mean annual direct healthcare costs per patient were low compared with that of other conditions treated in general practice. For example, the study by Redekop et al.,^28^ which included questionnaire-based data from 29 GP practices on the medical consumption of Dutch patients with diabetes mellitus, found that the average annual healthcare cost for GP-guided care for patients with diabetes mellitus was €600 per patient per year, making care for patients with diabetes mellitus approximately 6 times more expensive.

Our findings seem to suggest some level of agreement between clinical recommendations and clinical practice in the Netherlands.^8^ To illustrate, in accordance with the Dutch guidelines for low back pain and hand or wrist pain, relatively few patients with low back pain and relatively many patients with hand or wrist complaints were referred to secondary care facilities and diagnostic imaging.^9,10^ This seems to suggest that Dutch GPs largely adhere to existing GP guidelines, but further research is needed to confirm this.

Although the accurate calculation of population-based estimates requires sample weights, a gross estimate of the total costs of GP-guided care for patients with musculoskeletal complaints can be put at €301.3 million for the total Dutch population (ie, €39,180,531/13 × 100). This is slightly less than a cost estimate reported by the Dutch Ministry of Health, Welfare, and Sports (ie, €342.5 million).^29^ The difference in costs is probably because the Ministry's estimates included a slightly broader range of healthcare resources, but probably most importantly because of its use of tariffs to value healthcare utilization. Tariffs are typically higher than the standard prices used in this study and are generally discouraged by health economic guidelines, such as the Dutch guideline for costing studies, because they can differ extensively across healthcare providers and insurers and do not represent opportunity costs because of the regulated nature of the healthcare market.^13,22^

Our findings also showed that in GP-guided care, average primary care referral costs were higher than average secondary care referral costs (ie, €303 vs €155). This contrasts with how primary and secondary care compare in the total cost of musculoskeletal care, where hospital and medical specialist care make up almost half of the total cost (ie, €2710 of €6569 million).^29^ This difference is partly due to the way healthcare costs were allocated. For primary care referrals, costs were allocated based on treatment episodes (because a GP typically requests treatment in these cases). For secondary care referrals, costs were allocated based on a single consultation (and did not include costs related to diagnostic imaging, hospitalization, and surgery because a GP typically requests a consultation rather than follow-up examinations, surgery, or possible admissions).

Similar studies predicting having higher direct healthcare costs of GP-guided care are lacking. Nonetheless, a retrospective cohort study^18^ using Medical Expenditure Panel Survey data showed that various nonmodifiable factors were associated with having higher healthcare costs in patients with musculoskeletal pain. These factors included age, diagnosis type, and number of musculoskeletal diagnoses, which is in line with our findings. However, the retrospective cohort study also found several modifiable factors to be associated with having higher healthcare costs, such as a higher number of missed workdays, and greater pain interference, whereas higher self-reported physical and mental health were associated with lower healthcare costs. Unfortunately, we were not able to include these modifiable factors in our analysis because we used clinical registration data that did not include this information. Another study by Killingmo et al.,^14^ on modifiable prognostic factors of high healthcare costs among older people seeking primary care because of back pain, using data from 2 cohort studies, reported that a higher level of pain severity, disability, depression, and a lower level of physical health-related quality of life were associated with having higher healthcare costs. Again, because the data used in our study were collected for clinical purposes only, information on these types of modifiable prognostic factors was scarce and therefore only partially included in our analysis.

### 4.3. Strengths and limitations

This study was the first to provide an overview of healthcare utilization and direct healthcare costs of GP-guided care, stratified by specific regions and musculoskeletal conditions. There are several limitations of our study because of the use of clinical registration data. First, the registration of ICPC codes and healthcare utilization is generally of suboptimal quality due to issues with accuracy, concordance, and plausibility, leading to validity issues.^32^ To partially account for this, average cost estimates were not reported by ICPC codes but at a more aggregate level (ie, region of complaint and type of complaint), and furthermore, in the case of secondary care referrals, we were also able to partially correct for this by not including referrals that are clearly unrelated to a musculoskeletal complaint (eg, ophthalmologist referral). However, for referrals to primary care and imaging, this distinction could not be made based on the data available in the data set. Second, incomplete registration may have led to missing values in some of the variables.^32^ Although the amount of missing data was relatively low in our sample (ie, 93% of the observations were complete cases), data were imputed using information from observed data using MICE.^19^ Third, data availability is dependent on patients being registered at a GP clinic that is connected to the PHARMO Database. This means that data may be less available for younger patients or patients changing GP. However, many Dutch people have the same GP as an adult as they did in their childhood. This means that a complete medical history of younger patients is also available since the time of inception of the database and data availability is not related to being eligible for inclusion. Moreover, very few patients change GP. A report by the Patients' Federation shows that only 3% of all patients change GP each year. Therefore, the risk for potential bias for those who are older and those in the database for a longer time is probably very small. Forth, data on societal cost categories, such as patient costs and costs due to productivity losses from paid and unpaid work, could not be included in the current cost estimates. Fifth, a restricted number of available predictive factors in combination with a relatively low level of variation in both the predictive factors as well as the outcome variable (ie, direct healthcare costs) led to the low predictive performance of the regression model with statistically significant, but relatively small betas. However, in a large population, the budget impact can be high despite the betas being small. To illustrate, based on the model's estimates, women have—on average—€6 higher healthcare costs than men. As our sample included 227,101 women resulting in a total cost difference of €1,378,503 (ie, €6 * 227,101), equaling roughly €10,603,869 in the total Dutch population. Sixth, the use of stepwise selection methods based on significance level is suboptimal for predictive models. However, a recent overview on the selection of variables in the study by Sauerbrei et al.^31^ showed that there is not yet enough evidence on which to base recommendations for the selection of variables in multivariable analysis. Therefore, we used stepwise selection as this method already starts with a plausible model. Finally, this study provides an overview of the healthcare utilization and direct healthcare cost of GP-guided care among Dutch patients with MSK conditions, based on a highly representative sample of 13% of all adult patients registered with a Dutch GP practice (n = 16,479,000). Please note, however, that the current cost estimates cannot be interpreted as a proxy of the total (primary) healthcare costs of musculoskeletal conditions in the Netherlands because only the cost of care guided or referred to by GPs was included and, for example, hospitalization, surgeries, and physiotherapy visits without a GP referral were not included.

## 5. Conclusion

Most patients had a single consultation and the mean annual direct healthcare costs of GP-guided care in patients with a musculoskeletal condition was relatively low. Healthcare costs did not differ considerably across specific conditions. A key cost driver of GP-guided care in patients with musculoskeletal conditions was GP consultations, followed by referrals to primary care (including consultations in primary care). The investigated set of predictive factors explained a negligible part of the variance in direct healthcare costs. Thus, it is unclear which factors do explain high direct healthcare costs in patients with musculoskeletal complaints.

## Conflict of interest statement

The authors have no conflicts of interest to declare.

## Supplementary Material

SUPPLEMENTARY MATERIAL

## Appendix A. Supplemental digital content

Supplemental digital content associated with this article can be found online at http://links.lww.com/PAIN/B906.
