# Supplementary material for: Predicting direct healthcare costs of general practitioner–guided care in patients with musculoskeletal complaints
Source: Pain. 2023 Aug 15;165(2):404–11. doi: 10.1097/j.pain.0000000000003028 (PMC10785053; doi:10.1097/j.pain.0000000000003028)
Supplement: SUPPLEMENTARY MATERIAL [file jop-165-404-s001.pdf]

APPENDIX I. INCLUDED ICPC CODES

ICPC code	Description	Total 403,719 (n, %)	Region	Type of compliant
L01	Neck symptoms/complaints [ex. N01]	17,036 (4.2)	1	0
L02	Back symptoms/complaints	21,076 (5.2)	1	0
L03	Low back pain without radiation [ex. L86]	20,976 (5.2)	1	1
L04	Chest symptoms/complaints	26,652 (6.6)	0	0
L05	Flank symptoms/complaints	3,889 (1.0)	0	0
L06	Armpit symptoms/problems	1,398 (0.3)	0	0
L07	Jaw (joint) symptoms/complaints	4,291 (1.1)	0	0
L08	Shoulder symptoms/problems	25,317 (6.3)	2	8
L09	Arm symptoms/problems	6,852 (1.7)	2	0
L10	Elbow symptoms/problems	4,048 (1.0)	2	0
L11	Wrist symptoms/problems	7,260 (1.8)	2	0
L12	Hand/finger symptoms/conditions	18,346 (4.5)	2	0
L13	Hip symptoms/conditions	9,986 (2.5)	3	0
L14	Leg/thigh symptoms/conditions	17,478 (4.3)	3	0
L15	Knee symptoms/conditions	24,130 (6.0)	3	0
L16	Ankle symptoms/conditions	5,635 (1.4)	3	0
L17	Foot/toe symptoms/problems	25,840 (6.4)	3	0
L17.01	Metatarsalgia	967 (0.2)	3	0
L18	Muscle pain	6,633 (1.6)	0	0
L18.01	Fibromyalgia	386 (0.1)	0	0
L19	Symptoms multiple/nonspecific muscles	4,353 (1.1)	0	0
L20	Symptoms multiple/unspecified joints	4,087 (1.0)	0	0
L26	Fear of musculoskeletal cancer	10 (0.0)	0	0
L27	Fear of other musculoskeletal disease	98 (0.0)	0	0
L28	Musculoskeletal function limitation/disability	508 (0.1)	0	0
L29	Other complaints musculoskeletal system	4,137 (1.0)	0	0
L44	Preventive medication	75 (0.0)	0	0
L49	Prevention	30 (0.0)	0	0
L49.01	Fracture prevention	98 (0.0)	0	11
L70	Infectious musculoskeletal disease	90 (0.0)	0	0
L70.01	Osteomyelitis	50 (0.0)	0	0
L70.02	Septic arthritis	30 (0.0)	0	9
L71	Neoplasm musculoskeletal system	140 (0.0)	0	0
L71.01	Musculoskeletal malignancy	33 (0.0)	0	0
L71.02	Benign musculoskeletal neoplasm	117 (0.0)	0	0
L72	Fracture of radius/ulna	2,613 (0.6)	2	0
L73	Fracture of tibia/fibula	1,730 (0.4)	3	0
L74	Hand/foot fracture	2,932 (0.7)	0	0

L74.01	Fracture ossa phalanges hand	1,014 (0.3)	2	0
L74.02	Fracture ossa phalanges foot	1,107 (0.3)	3	0
L75	Fracture femur	785 (0.2)	3	0
L75.01	Fracture collum femoris	630 (0.2)	3	0
L76	Other fracture	930 (0.2)	0	0
L76.01	Fracture skull	125 (0.0)	0	0
L76.02	Fracture of nose	280 (0.1)	0	0
L76.03	Clavicle fracture	650 (0.2)	2	0
L76.04	Humerus fracture	860 (0.2)	2	0
L76.05	Rib fracture	786 (0.2)	0	0
L76.06	Fracture of vertebral column	716 (0.2)	1	0
L76.07	Pelvic fracture	253 (0.1)	3	0
L76.08	Fracture patella	93 (0.0)	3	0
L77	Sprain/distortion of ankle	7,284 (1.8)	3	5
L78	Knee sprain/distortion	3,836 (1.0)	3	4
L79	Other sprain/distortion	999 (0.2)	0	0
L79.01	Whiplash trauma to cervical spine	175 (0.0)	1	0
L80	Luxation/subluxation	473 (0.1)	0	0
L80.01	(Sub)luxation shoulder	401 (0.1)	2	0
L80.02	(Sub)luxation jaw joint	55 (0.0)	0	0
L80.03	(Sub)luxation finger	173 (0.0)	2	0
L80.04	(Sub)luxation acromio-clavicular joint	163 (0.0)	2	0
L80.05	(Sub)luxation radius head/sun arm	7 (0.0)	2	0
L81	Other musculoskeletal injuries	12,785 (3.2)	0	0
L81.01	Coup de fouet / whiplash	1,642 (0.4)	0	0
L81.02	Rib contusion	4,016 (1.0)	0	0
L82	Congenital musculoskeletal abnormality(s)	147 (0.0)	0	0
L82.01	Congenital hip luxation/hip dysplasia	29 (0.0)	0	0
L82.02	Spina bifida occulta	2 (0.0)	0	0
L82.03	Neck rib	4 (0.0)	0	0
L82.04	Clubfoot	13 (0.0)	0	0
L83	Syndrome cervical spine	1,645 (0.4)	1	0
L83.01	Hernia cervicalis	364 (0.1)	1	0
L84	Osteoarthritis/spondylosis vertebral column	927 (0.2)	1	0
L84.01	Osteoarthritis/spondylosis	571 (0.1)	1	0
L84.02	Spondylolysis/listhesis	53 (0.0)	1	0
L85	Acquired disorder(s) of the spine	197 (0.0)	1	0
L85.01	Scoliosis	259 (0.1)	1	0
L86	Low back pain with radiation	9,989 (2.5)	1	2
L86.01	HNP (thoracic/lumbar)	1,452 (0.4)	1	0
L87	Ganglion joint/tendon	3,962 (1.0)	2	7

L88	Rheumatoid arthritis/related condition(s)	691 (0.2)	0	9
L88.01	Rheumatoid arthritis	236 (0.1)	0	0
L88.02	Morbus Bechterew (ankylopoetic spondylitis)	75 (0.0)	0	0
L89	Cox osteoarthritis	2,899 (0.7)	3	0
L90	Gonarthrosis	4,769 (1.2)	3	3
L91	Other osteoarthritis/related disease	4,045 (1.0)	2	7
L92	Shoulder syndrome/PHS	9,262 (2.3)	2	8
L93	Epicondylitis lateralis	6,098 (1.5)	2	6
L94	Osgood-Schlatter/other osteochondropathy	51 (0.0)	0	0
L94.01	Osteochondritis dissecans	6 (0.0)	0	0
L94.02	Osgood-Schlatter's disease	18 (0.0)	0	0
L94.03	Epiphysiolysis femoral head	2 (0.0)	0	0
L94.04	Legg-Calvé-Perthes disease	3 (0.0)	0	0
L95	Osteoporosis	1,245 (0.3)	0	11
L95.01	Osteopenia	1,183 (0.3)	0	0
L95.02	Osteoporosis	352 (0.1)	0	0
L96	Acute meniscus/knee ligament injury	845 (0.2)	3	4
L96.01	Meniscus tear (lat./med.)	626 (0.2)	3	4
L96.02	Lig. cruciata injury (anterior/posterior)	187 (0.0)	3	4
L96.03	Collateral knee ligament injury	173 (0.0)	3	4
L96.04	Combined injury menisci/lig.cruciata/collateral/capillary ligament	33 (0.0)	3	4
L97	Chronic internal trauma to knee	287 (0.1)	3	3
L97.01	Old meniscus injury	41 (0.0)	3	3
L97.02	Non-traumatic meniscal defect	42 (0.0)	3	3
L97.03	Unstable knee	27 (0.0)	3	3
L97.04	Corpus liberum knee	11 (0.0)	3	3
L98	Acquired abnormality(s) extremities	562 (0.1)	0	0
L98.01	Mallet finger	651 (0.2)	2	7
L98.02	Pes planus	817 (0.2)	3	0
L98.03	Hallux valgus	1,517 (0.4)	3	0
L98.04	Hammer toe	315 (0.1)	3	0
L98.05	Leg length difference	92 (0.0)	3	0
L99	Other musculoskeletal disease(s)	7,993 (2.0)	0	0
L99.01	Bursitis [ex. L92]	6,546 (1.6)	0	0
L99.02	Tendovaginitis/tendinitis	6,297 (1.6)	2	7
L99.03	Dupuytren's contracture	1,273 (0.3)	2	7
L99.04	Trigger finger	2,800 (0.7)	2	7
L99.05	Epicondylitis medialis	729 (0.2)	2	6
L99.06	Tietze's syndrome	1,030 (0.3)	0	0
L99.07	Retropatellar chondropathy/patellofemoral syndrome	975 (0.2)	3	3
L99.08	Heel spur/plantar fasciitis	5,017 (1.2)	3	0

L99.09	Hyperlaxity	54 (0.0)	0	0
L99.10	Corpus liberum joint [ex. L97]	6 (0.0)	0	0
L99.11	Pseudarthrosis	7 (0.0)	0	0
L99.12	Polymyalgia rheumatica	520 (0.1)	0	10
L99.13	Artritis psoriatica	53 (0.0)	0	0
N93	Carpal tunnel syndrome	4,079 (1.0)	2	7

Region: 0= others; 1= spine; 2= upper extremity; 3=lower extremity

Type of complaint: 0= none; 1= Non-specific low back pain; 2= Lumbosacral radicular syndrome; 3= Non-traumatic knee pain; 4= Traumatic knee pain; 5= Ankle ligament injury; 6= Epicondylitis; 7= Hand and wrist pain; 8= Shoulder pain; 9= Arthritis; 10= Polymyalgia rheumatica and arteritis temporalis; 11= Fracture prevention.

APPENDIX II. PREDICTIVE VARIABLES

Comorbidities	
Condition	ICPC codes included
Depression	P03 Feeling depressed<br>P76 Depressive disorder
Cardiovasculair disease	K74 Ischaemic heart disease w. angina<br>K75 Acute myocardial infarction<br>K76 Ischaemic heart disease w/o angina<br>K77 Heart failure<br>K85 Elevated blood pressure<br>K86 Hypertension uncomplicated<br>K87 Hypertension complicated<br>K89 Transient cerebral ischaemia<br>K90 Stroke/cerebrovascular accident<br>K92 Atherosclerosis/PVD<br>K99 Cardiovascular disease other<br>T 93 Lipid disorder
DM	T90.1Diabetes insulin dependent<br>T90.2 Diabetes non-insulin dependent
Smoking	P17 Tobacco abuse
Obese	T82 Obesity (QI ≥30)<br>T83 Overweight (27≤ QI <30)
SES	
	The variable SES was determined as a relative measure based on the scores of the Netherlands Institute for Social Research (SCP score). This SCP score is available for all 4-digit postal codes with more than 100 households and is provided every 4 years by the Netherlands Institute for Social Research since 1995. The score is based on mean household income, percentage of households with a low income, percentage of inhabitants without a paid job, and percentage of households with a low mean education. This information is obtained via phone calls from the organization Evers Direct Marketing Besloten Vennootschap (EDM-BV) to 1 person in each 6-digit postal code (usually 1 street) and aggregated to 4-digit postal codes. To determine 4 categories of SES in our cohort (low, middle, high, unknown), we assigned the most recent SCP scores to all patients in the PHARMO Database Network and used tertiles as cutpoints for classification to low, middle and high. Patients without an SCP code available were classified as unknown.

APPENDIX III. BASELINE CHARACTERISTICS PER REGION

Spine					
	**Overall (n= 74553)**	**Q1/Q2* (n=37,277)**	**Q3 (n=18,638)**	**Q4 (n=18,638)**	**Top 5% HCU (n=3,728)**
Gender; female (Yes; n, %)	42,451 (56.9)	20,791 (55.8)	10,663 (57.2)	10,997 (59.0)	2,187 (58.7)
Age (mean, SD)	51.47 (18.23	50.89 (18.20)	51.44 (18.18)	52.64 (18.29)	53.19 (18.51)
**Comorbidities**					
Chronic Disease Score (mean, SD)	2.63 (3.62)	2.38 (3.46)	2.97 (3.70)	2.97 (3.81)	3.13 (3.89)
Number of MSK diagnosis (mean, SD)	0.07 (0.30)	0.07 (0.29)	0.08 (0.31)	0.08 (0.30)	0.08 (0.31)
Depression (Yes; n, %)	431 (0.6)	202 (0.5)	118 (0.6)	111 (0.6)	20 (0.5
Obese (Yes; n, %)	231 (0.3)	93 (0.2)	81 (0.4)	57 (0.3)	18 (0.5)
Smoking (Yes; n, %)	321 (0.4)	137 (0.4)	108 (0.6)	76 (0.4)	15 (0.4)
Cardiovascular (Yes; n, %)	19,776 (26.5)	9,334 (25.0)	5,115 (27.4	5,327 (28.6)	1,107 (29.7)
Diabetes (Yes; n, %)	3,989 (5.4)	1,767 (4.7)	1,082 (5.8)	1,140 (6.1)	240 (6.4)
**Social Economic Status**					
Low (n, %)	27,099 (36.3)	14,258 (38.2)	5,810 (31.2)	7,031 (37.7)	1,379 (37.0)
Middle (n, %)	24,238 (32.5)	12,535 (33.6)	6,119 (32.8)	5,584 (30.0)	1,026 (27.5)
High (n, %)	23,216 (31.1)	10,484 (28.1)	6,709 (36.0)	6,023 (32.3)	1,323 (35.5)
**Upper extremity**					
	**Overall (n=109,202)**	**Q1/Q2 (n=57,007)**	**Q3 (n=25,075)**	**Q4 (n=27,120)**	**Top 5% HCU (n=5552)**
Gender; female (Yes; n, %)	60,762 (55.6)	31,223 (54.8)	13,931 (55.6)	15,608 (57.6)	3,282 (59.1)
Age (mean, SD))	52.68 (17.49)	52.01 (17.66)	53.04 (17.51)	53.78 (17.03)	54.17 (16.41)
**Comorbidities**					
Chronic Disease Score (mean (SD))	2.68 (3.63)	2.46 (3.51)	2.84 (3.69)	2.98 (3.79)	3.07 (3.81)
Number of MSK diagnosis (mean (SD))	0.08 (0.31)	0.08 (0.31)	0.08 (0.31)	0.09 (0.33)	0.08 (0.32)
Depression (Yes; n, %)	571 (0.5)	291 (0.5)	133 (0.5)	147 (0.5)	31 (0.6)
Obese (Yes; n, %)	335 (0.3)	156 (0.3)	90 (0.4)	89 (0.3)	20 (0.4)
Smoking (Yes; n, %)	479 (0.4)	220 (0.4)	130 (0.5)	129 (0.5)	28 (0.5)
Cardiovascular (Yes; n, %)	30,179 (27.6)	14,919 (26.2)	7,339 (29.3)	7,921 (29.2)	1,733 (31.2)
Diabetes (Yes; n, %)	6,178 (5.7)	2,908 (5.1)	1,549 (6.2)	1,721 (6.3)	383 (6.9)
**Social Economic Status**					
Low (n, %)	39,439 (36.1	21,306 (37.4)	7,953 (31.7)	10,180 (37.5)	2,278 (40.1)
Middle (n, %)	36,086 (33.0)	19,511 (34.2)	8,374 (33.4)	8,201 (30.2)	1,597 (28.1)
High (n, %)	33,677 (30.8)	16,190 (28.4)	8,748 (34.9)	8,739 (32.2)	1,800 (31.7)
**Lower extremity**					
	**Overall (n=113,449)**	**Q1/Q2* (n=56,756)**	**Q3 (n=28,338)**	**Q4 (n=28,355)**	**Top 5% HCU (n=5,675)**
Gender; female (Yes; n, %)	64,652 (57.0)	31,919 (56.2)	16,095 (56.8)	16,638 (58.7)	3,449 (60.8)
Age (mean, SD)	53.61 (19.07)	52.50 (19.26)	54.23 (19.41)	55.20 (18.19)	57.29 (17.48)

**Comorbidities**					
Chronic Disease Score (mean, SD)	2.91 (3.80)	2.65 (3.67)	3.21 (3.94)	3.14 (3.89)	3.33 (3.90)
Number of MSK diagnosis (mean, SD)	0.08 (0.31)	0.07 (0.30)	0.08 (0.31)	0.08 (0.32)	0.09 (0.34)
Depression (Yes; n, %)	584 (0.5)	298 (0.5)	152 (0.5)	134 (0.5)	27 (0.5)
Obese (Yes; n, %)	376 (0.3)	163 (0.3)	102 (0.4)	111 (0.4)	22(0.4)
Smoking (Yes; n, %)	413 (0.4)	186 (0.3)	119 (0.4)	108 (0.4)	19 (0.3)
Cardiovascular (Yes; n, %)	33,152 (29.2)	15,439 (27.2)	8,816 (31.1)	8,897 (31.4)	1,873 (33.0)
Diabetes (Yes; n, %)	6,711 (5.9)	3,163 (5.6)	1,798 (6.3)	1,750 (6.2)	372 (6.6)
**Social Economic Status**					
Low (n, %)	41,492 (36.6)	20,986 (37.0)	9,375 (33.1)	11,131(39.3)	2,278 (40.1)
Middle (n, %)	37,001 (32.6)	19,314 (34.0)	9,419 (33.2)	8,268 (29.2)	1,597 (28.1)
High (n, %)	34,956 (30.8)	16,456 (29.0)	9,544 (33.7)	8,956 (31.6)	1,800 (31.7)

**Q 1en Q2 are merged as many patients had the same cost and were therefore hard to disguise.*

APPENDIX IV. BASELINE CHARACTERISTICS PER TYPE OF COMPLAINT

**Hand/wrist**					
	**Overall (n=23,107)**	**Q1/Q2* (n=11,884)**	**Q3 (n=5,447)**	**Q4 (n=5,776)**	**Top 5% HCU (n=1,335)**
Gender; female (Yes; n, %)	13,925 (60.3)	7,127 (60.0)	3,242 (59.5)	3,556 (61.6)	836 (62.6)
Age (mean, SD)	56.69 (16.44)	55.94 (16.82)	57.85 (16.39)	57.14 (15.58)	55.72 (15.46)
**Comorbidities**					
Chronic Disease Score (mean, SD)	3.09 (3.79)	2.85 (3.67)	3.36 (3.91)	3.33 (3.87)	3.15 (3.77)
Number of MSK diagnosis (mean, SD)	0.09 (0.32)	0.08 (0.32)	0.08 (0.31)	0.09 (0.34)	0.09 (0.32)
Depression (Yes; n, %)	114 (0.5)	57 (0.5	23 (0.4)	34 (0.6)	6 (0.4)
Obese (Yes; n, %)	69 (0.3)	32 (0.3)	18 (0.3)	19 (0.3)	1 (0.1)
Smoking (Yes; n, %)	99 (0.4)	43 (0.4)	23 (0.4)	33 (0.6)	9 (0.7)
Cardiovascular (Yes; n, %)	7,631 (33.0)	3,756 (31.6)	1,946 (35.7)	1,929 (33.4)	449 (33.6)
Diabetes (Yes; n, %)	1,593 (6.9)	755 (6.4)	393 (7.2)	445 (7.7)	110 (8.2)
**Social Economic Status**					
Low (n, %)	8,361 (36.2)	4,482 (37.7)	1,778 (32.6)	2,101 (36.4)	509 (38.1)
Middle (n, %)	7,801 (33.8)	4,119 (34.7)	1,892 (34.7)	1,790 (31.0)	425 (31.8)
High (n, %)	6,945 (30.1)	3,283 (27.6)	1,777 (32.6)	1,885 (32.6)	401 (30.0)
**Knee**					
	**Overall (n=12,308)**	**Q1/Q2* (n=6,187)**	**Q3 (n=3,047)**	**Q4 (n=3,074)**	**Top 5% HCU (n=616)**
Gender; female (Yes; n, %)	6,471 (52.6)	2,846 (46.0)	1,678 (55.1	1,947 (63.3)	423 (68.7)
Age (mean, SD)	60.57 (16.13)	57.95 (16.80)	63.27 (15.73)	63.17 (14.16	63.41 (13.44)
**Comorbidities**					
Chronic Disease Score (mean, SD)	3.66 (4.02)	3.19 (3.88)	4.15 (4.12)	2.03 (2.97)	4.00 (4.07)
Number of MSK diagnosis (mean, SD)	0.09 (0.33)	0.08 (0.31)	0.09 (0.35)	0.10 (0.36)	0.12 (0.42)
Depression (Yes; n, %)	56 (0.5)	27 (0.4)	11 (0.4)	18 (0.6)	4 (0.6)
Obese (Yes; n, %)	43 (0.3)	19 (0.3)	11 (0.4)	13 (0.4)	4 (0.6)
Smoking (Yes; n, %)	45 (0.4)	28 (0.5)	13 (0.4)	4 (0.1)	0 (0.0)
Cardiovascular (Yes; n, %)	4,596 (37.3)	2,073 (33.5)	1,271 (41.7)	1,252 (40.7)	253 (41.1)
Diabetes (Yes; n, %)	967 (7.9)	416 (6.7)	253 (8.3)	298 (9.7)	63 (10.2)
**Social Economic Status**					
Low (n, %)	4,273 (34.7)	2,262 (36.6)	912 (29.9)	1,099 (35.8)	253 (41.1)
Middle (n, %)	4,372 (35.5)	2,227 (36.0)	1,161 (38.1)	984 (32.0)	169 (27.4)
High (n, %)	3,663 (29.8)	1,698 (27.4)	974 (32.0)	991 (32.2)	194 (31.5)
Referrals (mean, SD)	60.22 (123.54)	0.00 (0.00)	10.79 (22.32)	230.41 (148.09)	459.05 (143.19)
**Low Back Pain**					
	**Overall (n=20,976)**	**Q1/Q2* (n=10,750)**	**Q3 (n=4,982)**	**Q4 (n=5,244)**	**Top 5% HCU (n=1,061)**
Gender; female (n, %)	11,292 (53.8)	5,646 (52.5)	2,635 (52.9	3,011 (57.4)	621 (58.5)

Age (mean, SD)	50.96 (18.13)	50.90 (17.68)	50.26 (18.17)	51.74 (18.95)	52.92 (19.53)
**Comorbidities**					
Chronic Disease Score (mean, SD)	2.46 (3.51)	2.26 (3.34)	2.49 (3.46)	2.86 (3.82)	2.98 (3.74)
Number of MSK diagnosis (mean, SD)	0.06 (0.28)	0.06 (0.27)	0.07 (0.29)	0.07 (0.30)	0.07 (0.28)
Depression (Yes; n, %)	129 (0.6)	59 (0.5)	34 (0.7)	36 (0.7)	4 (0.4)
Obese (Yes; n, %)	60 (0.3)	27 (0.3)	19 (0.4)	14 (0.3)	7 (0.7)
Smoking (Yes; n, %)	91 (0.4)	36 (0.3)	33 (0.7)	22 (0.4)	4 (0.4)
Cardiovascular (Yes; n, %)	5,384 (25.7)	2,629 (24.5)	1,285 (25.8)	1,470 (28.0)	318 (30.0)
Diabetes (Yes; n, %)	1,090 (5.2)	502 (4.7)	263 (5.3)	325 (6.2)	74 (7.0)
**Social Economic Status**					
Low (n, %)	7,570 (36.1)	4,087 (38.0)	1,497 (30.0)	1,986 (37.9)	400 (37.7)
Middle (n, %)	6,968 (33.2)	3,725 (34.7)	1,661 (33.3)	1,582 (30.2)	306 (28.8)
High (n, %)	6,438 (30.7)	2,938 (27.3)	1,824 (36.6)	1,676 (32.0)	355 (33.5)
**Low Back Pain radicular pain**					
	**Overall (n=9,989)**	**Q1/Q2* (n=4,995)**	**Q3 (n=2,500)**	**Q4 (n=2,494)**	**Top 5% HCU (n=500)**
Gender; female (Yes; n, %)	5,722 (57.3)	2,789 (55.8)	1,472 (58.9)	1,461 (58.6)	312 (62.4)
Age (mean, SD)	55.03 (16.72)	54.34 (16.46)	56.15 (16.90)	55.29 (16.99)	54.27 (17.34)
**Comorbidities**					
Chronic Disease Score (mean, SD)	3.02 (3.78)	2.70 (3.61)	3.60 (4.04)	3.10 (3.78)	3.25 (3.88)
Number of MSK diagnosis (mean, SD)	0.08 (0.31)	0.07 (0.29)	0.09 (0.34)	0.08 (0.30	0.09 (0.29)
Depression (Yes; n, %)	49 (0.5)	19 (0.4)	14 (0.6)	16 (0.6)	1 (0.2)
Obese (Yes; n, %)	39 (0.4	15 (0.3)	14 (0.6)	10 (0.4)	3 (0.6)
Smoking (Yes; n, %)	51 (0.5)	30 (0.6)	11 (0.4)	10 (0.4)	5 (1.0)
Cardiovascular (Yes; n, %)	3,060 (30.6)	1,416 (28.3)	852 (34.1)	792 (31.8)	151 (30.2)
Diabetes (Yes; n, %)	637 (6.4)	305 (6.1)	170 (6.8)	162 (6.5	32 (6.4)
**Social Economic Status**					
Low (n, %)	3,402 (34.1)	1,724 (34.5)	802 (32.1)	876 (35.1)	186 (37.2)
Middle (n, %)	3,291 (32.9)	1,742 (34.9)	786 (31.4)	763 (30.6)	136 (27.2)
High (n, %)	3,296 (33.0)	1,529 (30.6)	912 (36.5)	855 (34.3)	178 (35.6)
**Shoulder**					
	**Overall (n=34,579)**	**Q1/Q2* (n=17,349)**	**Q3 (n=8,663)**	**Q4 (n= 8,567)**	**Top 5% HCU (n=1,734)**
Gender; female (Yes; n, %)	18,495 (53.5)	9,124 (52.6)	4,717 (54.4)	4,654 (54.3)	993 (57.3)
Age (mean, SD)	53.98 (16.82)	53.46 (16.89)	54.11 (17.35)	54.91 (16.09)	55.56 (15.87)
**Comorbidities**					
Chronic Disease Score (mean, SD)	2.78 (3.67)	2.52 (3.50)	3.06 (3.84)	3.00 (3.79)	3.18 (3.79)
Number of MSK diagnosis (mean, SD)	0.08 (0.31)	0.07 (0.30)	0.08 (0.32)	0.08 (0.33)	0.09 (0.34)
Depression (Yes; n, %)	189 (0.5)	85 (0.5)	51 (0.6)	53 (0.6)	16 (0.9)
Obese (Yes; n, %)	124 (0.4)	57 (0.3)	34 (0.4)	33 (0.4)	7 (0.4)
Smoking (Yes; n, %)	153 (0.4)	68 (0.4)	46 (0.5)	39 (0.5)	7 (0.4)

Cardiovascular (Yes; n, %)	9,841 (28.5)	4,721 (27.2)	2,603 (30.0)	2,517 (29.4)	545 (31.4)
Diabetes (Yes; n, %)	2117 (6.1)	986 (5.7)	606 (7.0)	525 (6.1)	124 (7.2)
**Social Economic Status**					
Low (n, %)	12,539 (36.3)	6,398 (36.9)	2,794 (32.3)	3,347 (39.1)	689 (39.7)
Middle (n, %)	11,603 (33.6)	6,072 (35.0)	2,913 (33.6)	2,618 (30.6)	511 (29.5)
High (n, %)	10,437 (30.2)	4,879 (28.1)	2,956 (34.1)	2,602 (30.4)	534 (30.8)
**Q 1en Q2 are merged as many patients had the same costs and were therefore hard to distinguish*					

APPENDIX V. COSTS PER REGION AND TYPE OF COMPLAINT

**Spine**					
	Overall (n= 74553)	Q1/Q2* (n=37,277)	Q3 (n=18,638)	Q4 (n=18,638)	Top 5% HCU (n=3,728)
Consultation costs (mean, SEM)	44 (0.08)	36 (0.00)	52 (0.15)	52 (0.28)	57 (0.83)
Medication costs (mean, SEM)	2 (0.08)	0 (0.00)	2 (0.04)	5 (0.32)	16 (1.49)
Referrals primary care costs (mean, SEM)	47 (0.39)	0 (0.00)	2 (0.08)	184 (1.07)	349 (1.07)
Referrals secondary care costs (mean, SEM)	13 (0.15)	0 (0.00)	3 (0.07)	51 (0.52)	98 (1.75)
Imaging costs (mean, SEM)	2 (0.05)	0 (0.00)	0 (0.02)	7 (0.20)	12 (0.61)
Total referral costs (mean, SEM)	62(0.47)	0 (0.00)	5 (0.11)	241 (1.12)	459 (2.81)
Total cost (mean, SEM)	93(0.49)	36 (0.00)	59 (0.14)	298 (1.13)	532 (2.91)
**Upper extremity**					
	Overall (n=109,202)	Q1/Q2* (n=57,007)	Q3 (n=25,075)	Q4 (n=27,120)	Top 5% HCU (n=5552)
Consultation costs (mean, SEM)	41 (0.05)	36 (0.00)	47 (0.10)	47 (0.18)	49 (0.45)
Medication costs (mean, SEM)	1 (0.04)	0 (0.00)	2 (0.03)	2 (0.15)	5 (0.71)
Referrals primary care costs (mean, SEM)	30 (0.25)	0 (0.00)	1 (0.05)	121 (0.79)	270 (1.91)
Referrals secondary care costs (mean, SEM)	17 (0.13)	0 (0.00)	4 (0.08)	65 (0.42)	94 (1.38)
Imaging costs (mean, SEM)	3 (0.05)	0 (0.00)	0 (0.02)	12 (0.19)	17 (0.56)
Total referral costs (mean, SEM)	50 (0.32)	0 (0.00)	6 (0.09)	198 (0.80)	380 (1.89)
Total cost (mean, SEM)	93 (0.33)	36 (0.00)	55 (0.09)	247 (0.78)	434 (1.93)
**Lower extremity**					
	Overall (n=113,449)	Q1/Q2* (n=56,756)	Q3 (n=28,338)	Q4 (n=28,355)	Top 5% HCU (n=5,675)
Consultation costs (mean, SEM)	43 (0.07)	36 (0.00)	53 (0.14)	48 (0.20)	54 (0.62)
Medication costs (mean, SEM)	1 (0.03)	0 (0.00)	1 (0.03)	2 (0.10)	4 (0.43)
Referrals primary care costs (mean, SEM)	35 (0.26)	0 (0.00)	4 (0.08)	135 (0.78)	274 (1.99)
Referrals secondary care costs (mean, SEM)	21 (0.15)	0 (0.00)	10 (0.12)	75 (0.44)	119 (1.40)
Imaging costs (mean, SEM)	5 (0.06)	0 (0.00)	2 (0.05)	17 (0.22)	24 (0.64)
Total referral costs (mean, SEM)	61 (0.35)	0 (0.00)	16 (0.16)	227 (0.77)	417 (1.94)
Total cost (mean, SEM)	105 (0.36)	36 (0.00)	70 (0.15)	277 (0.77)	475 (1.88)
**Hand/wrist**					
	Overall (n=23,107)	Q1/Q2* (n=11,884)	Q3 (n=5,447)	Q4 (n=5,776)	Top 5% HCU (n=1,335)
Consultation costs (mean, SEM)	41 (0.11)	36 (0.00)	45 (0.21)	46 (0.36)	45 (0.77)
Medication costs (mean, SEM)	1 (0.05)	0 (0.01)	3 (0.06)	2 (0.21)	3 (0.79)
Referrals primary care costs (mean, SEM)	23 (0.46)	(0.00)	1 (0.09)	91 (1.54)	243 (3.39)
Referrals secondary care costs (mean, SEM)	23 (0.34)	0 (0.00)	7 (0.21)	87 (0.92)	91 (2.87)
Imaging costs (mean, SEM)	2 (0.08)	0 (0.00)	0 (0.03)	8 (0.32)	11 (0.80)
Total referral costs (mean, SEM)	48 (0.65)	0 (0.00)	8 (0.23)	185 (1.53)	346 (3.12)

Total cost (mean, SEM)	90 (0.66)	36 (0.01)	56 (0.22)	232 (1.49)	394 (3.10)
**Knee**					
	Overall (n=12,308)	Q1/Q2* (n=6,187)	Q3 (n=3,047)	Q4 (n=3,074)	Top 5% HCU (n=616)
Consultation costs (mean, SEM)	45 (0.23)	36 (0.00)	52 (0.40)	54 (0.79)	61 (2.47)
Medication costs (mean, SEM)	2 (0.07)	0 (0.02)	3 (0.12)	4 (0.23)	4 (0.48)
Referrals primary care costs (mean, SEM)	33 (0.80)	0 (0.00)	2 (0.18)	130 (2.45)	293 (6.00)
Referrals secondary care costs (mean, SEM)	24 (0.51)	0 (0.00)	8 (0.34)	89 (1.48)	147 (4.63)
Imaging costs (mean, SEM)	3 (0.14)	0 (0.00)	1 (0.08)	11 (0.54)	18 (1.60)
Total referral costs (mean, SEM)	60 (1.11)	0 (0.00)	11 (0.40)	230 (2.67)	459 (5.77)
Total costs (mean, SEM)	107 (1.16)	36.89 (0.02)	66 (0.42)	288 (2.63)	524 (5.56)
**Low Back Pain**					
	Overall (n=20,976)	Q1/Q2* (n=10,750)	Q3 (n=4,982)	Q4 (n=5,244)	Top 5% HCU (n=1,061)
Consultation costs (mean, SEM)	43 (0.13)	36 (0.00)	49 (0.22)	50 (0.44)	53 (1.03)
Medication costs (mean, SEM)	2 (0.11)	0 (0.01)	2 (0.06)	4 (0.44)	10 (2.01)
Referrals primary care costs (mean, SEM)	46 (0.74)	0 (0.00)	0 (0.00)	182 (2.01)	354 (4.80)
Referrals secondary care costs (mean, SEM)	9 (0.22)	0 (0.00)	1 (0.07)	36 (0.78)	68 (2.61)
Imaging costs (mean, SEM)	1 (0.09)	0 (0.00)	0 (0.03)	5 (0.34)	10 (1.15)
Total referral costs (mean, SEM)	56 (0.85)	0 (0.00)	1 (0.07)	224 (2.11)	431 (5.04)
Total cost (mean, SEM)	101 (0.88)	36 (0.01)	52 (0.17)	278 (2.07)	495 (5.05)
**Low Back Pain radicular pain**					
	Overall (n=9,989)	Q1/Q2* (n=4,995)	Q3 (n=2,500)	Q4 (n=2,494)	Top 5% HCU (n=500)
Consultation costs (mean, SEM)	46 (0.26)	38 (0.06)	58 (0.61)	52 (0.74)	57 (1.86)
Medication costs (mean, SEM)	4 (0.34)	1 (0.03)	4 (0.24)	10 (1.32)	31 (6.19)
Referrals primary care costs (mean, SEM)	58 (1.18)	0 (0.00)	11 (0.57)	220 (2.85)	374 (7.68)
Referrals secondary care costs (mean, SEM)	26 (0.60)	0 (0.00)	32 (0.92)	74 (1.85)	144 (5.78)
Imaging costs (mean, SEM)	2 (0.14)	0 (0.00)	1 (0.13)	7 (0.53)	12 (1.49)
Total referral costs (mean, SEM)	86 (1.49)	0 (0.00)	45 (1.11)	301 (3.03)	530 (7.19)
Total cost (mean, SEM)	137 (1.58)	39 (0.06)	108 (0.94)	364 (3.24)	619 (8.17)
**Shoulder**					
	Overall (n=34,579)	Q1/Q2* (n=17,349)	Q3 (n=8,663)	Q4 (n= 8,567)	Top 5% HCU (n=1,734)
Consultation costs (mean, SEM)	43 (0.11)	36 (0.00)	52 (0.24)	47 (0.33)	51 (0.96)
Medication costs (mean, SEM)	2 (0.11)	1 (0.01)	3 (0.07)	4 (0.43)	8 (2.09)
Referrals primary care costs (mean, SEM)	48 (0.57)	0 (0.00)	6 (0.22)	188 (1.48)	331 (3.70)
Referrals secondary care costs (mean, SEM)	17 (0.24)	0 (0.00)	8 (0.20)	60 (0.79)	108 (2.47)
Imaging costs (mean, SEM)	2 (0.08)	0 (0.00)	0 (0.04)	7 (0.30)	13 (0.91)
Total referral costs (mean, SEM)	67 (0.70)	0 (0.00)	14 (0.31)	255 (1.48)	453 (3.74)
Total cost (mean, SEM)	112 (0.71)	37 (0.01)	69 (0.30)	305 (1.51)	512 (3.99)

**Q 1en Q2 are merged as many patients had the same costs and were therefore hard to distinguish.*
Costs are presented in euros 2021.

APPENDIX VI. REGRESSION MODEL

Total healthcare costs = 92.43006 + 1.979414*number of MSK diagnosis -18.14823*region none + 3.684978* region spine - 10.80206*region upper extremity - 9.017858* SES middle -1.360714*SES high + 0.827822* CDS + 6.073372* female + 0.171268* age				
	Regression Coefficient (SE)	Pooled Standard Error	Pooled 95% CI	
			2.5 %	97.5 %
Intercept	92.43006	1.127535	90.22009	94.64003
Age	0.171268	0.01688466	0.1381741	0.2043619
Female	6.073372	0.5544197	4.986709	7.160035
Chronic Disease Score	0.827822	0.1122947	0.6077244	1.04792
Number of MSK diagnosis	1.979414	0.6793149	0.6479568	3.310871
Region (ref: lower extremity)				
Region none*	-18.14823	0.6575838	-19.43709	-16.85937
Region spine	3.684978	0.6749788	2.36202	5.007936
Region upper extremity	-10.80206	0.7413566	-12.25512	-9.349001
SES (ref: SES low)				
SES middle	-9.017858	0.3258425	-9.656509	-8.379207
SES high	-1.360714	0.6316623	-2.598772	-0.1226559
*This includes a musculoskeletal complaint that are not restricted to one region such as fibromyalgia, osteoporosis, and rheumatoid arthritis. R^2 = 0.007, RMSE = 127.8				
